# Frequency and antimicrobial resistance patterns of bacteria implicated in community urinary tract infections: a ten-year surveillance study (2000–2009)

**DOI:** 10.1186/1471-2334-13-19

**Published:** 2013-01-18

**Authors:** Inês Linhares, Teresa Raposo, António Rodrigues, Adelaide Almeida

**Affiliations:** 1 Department of Biology and CESAM, University of Aveiro, 3810-193, Aveiro, Portugal; 2 Clinical Analysis Laboratory Avelab, Rua Cerâmica do Vouga, 3800-011, Aveiro, Portugal

**Keywords:** Community-acquired urinary tract infection, Uropathogens, Antibiotics, Antimicrobial resistance, Multidrug resistance

## Abstract

**Background:**

Urinary tract infection (UTI) is one of the most common infectious diseases at the community level. In order to assess the adequacy of the empirical therapy, the prevalence and the resistance pattern of the main bacteria responsible for UTI in the community (in Aveiro, Portugal) was evaluated throughout a ten-year period.

**Methods:**

In this retrospective study, all urine samples from patients of the District of Aveiro, in ambulatory regime, collected at the Clinical Analysis Laboratory Avelab during the period 2000–2009 were analysed. Samples with more than 10^5^ CFU/mL bacteria were considered positive and, for these samples, the bacteria were identified and the profile of antibiotic susceptibility was characterized.

**Results:**

From the 155597 samples analysed, 18797 (12.1%) were positive for bacterial infection. UTI was more frequent in women (78.5%) and its incidence varied with age, affecting more the elderly patients (38.6%). Although *E. coli* was, as usual, the most common pathogen implicated in UTI, it were observed differences related to the other bacteria more implicated in UTI relatively to previous studies. The bacteria implicated in the UTI varied with the sex of the patient, being *P. aeruginosa* a more important cause of infection in men than in women. The incidence of the main bacteria changed over the study period (*P. aeruginosa*, *Klebsiella spp* and *Providencia spp* increased and *Enterobacter spp* decreased). Although *E. coli* was responsible for more than an half of UTI, its resistance to antibiotics was low when compared with other pathogens implicated in UTI, showing also the lowest percentage of multidrug resistant (MDR) isolates (17%). Bacteria isolated from females were less resistant than those isolated from males and this difference increased with the patient age.

**Conclusions:**

The differences in sex and age must be taken into account at the moment of empirical prescription of antimicrobials. From the recommended antimicrobials by the European Association of Urology guidelines, the first line drugs (pivmecillinam and nitrofurantoin) and the alternative antibiotic amoxicillin-clavulanic acid (AMX-CLA) are appropriate to treat community-acquired UTI, but the fluoroquinolones should not be suitable to treat male infections and the trimethoprim-sulfamethoxazole (SXT) shall not be used in the treatment of UTI at this level.

## Background

The antimicrobials misuse in clinical medicine has led to an increase of the microbial resistance and the consequent spread of bacterial resistant strains is a serious public health problem. Urinary tract infection (UTI) is one the most common infectious diseases of the community and also of the hospital settings, resulting in high rates of morbidity and high economic costs associated with its treatment [1-3]. Uncomplicated UTI occurs in patients without any anatomic or functional abnormality in the urinary tract and may reach, on average, 6.1 days of symptoms, 2.4 days of restricted activity and 0.4 bed days [4-6]. Uncomplicated cystitis (infection of bladder) is the most common UTI and is responsible for 95% of all symptomatic urinary tract infection [7].

Some studies carried out in the community have shown that uropathogens such as *Escherichia coli* (46.4 - 74.2%), *Klebsiella spp* (6.0 - 13.45%), *Proteus spp* (4.7 - 11.9%) and *Enterococcus spp* (5.3 - 9.54%) represent the main causes of UTI [2,8-16]. *E. coli* has been indicated as the most frequent uropathogen involved in the community-acquired UTI [10,13,16,17] due to the fact of belonging to the normal flora of the human intestine and therefore easily colonizing the urinary tract. Some strains of *E. coli* isolated from sexually active patients matched with faecal isolates from their partners, which indicate that the ITU can be sexually transmitted [18]. Community-acquired urinary tract infections are mainly uncomplicated, colonizing preferably the bladder and causing cystitis [18,19]. However, *E. coli* may ascend through the ureters to the kidneys and cause more severe infections such as pyelonephritis [18,19]. The bacterium *Pseudomonas aeruginosa* is emerging as an opportunistic pathogen of UTI in the community and has been associated to 10.7 - 25% of cases [3,10,11,20-22].

The early treatment of UTI decreases the rate of morbidity, implying that in most cases antimicrobial therapy be prescribed empirically [10]. In order to administer an appropriate empirical therapy it is crucial to know the main bacteria usually involved in the urinary tract infection as well as their respective antimicrobial resistance pattern [15,23]. This procedure allows controlling the increase of antimicrobial resistance and the spread of resistant bacterial strains that represent a public health problem worldwide.

The treatment of acute uncomplicated cystitis recommended by the guidelines of the European Association of Urology (EAU) include fosfomycin, trometamol, pivmecillinam (a penicillin), nitrofurantoin (a nitrofuran) as first-line therapy and, as an alternative therapy, fluoroquinolones, cepodoxime proxetil, the sulfonamides SXT and trimethoprim, if the local resistance is less than 20% [24,25]. These recommendations for UTI empiric treatment should be adjusted taking into account the geographical location of the patient, age, sex and other diseases [26]. According the ARESC, an international survey on the antimicrobial resistance of pathogens implicated in uncomplicated UTIs [27], *E. coli* showed high resistance to the sulfonamide SXT (29.4%) and reduce resistance to nitrofurantoin (1.6%) and to fluoroquinolone ciprofloxacin (8.1%) in nine European countries and in Brazil.

Unfortunately there are few publications about the main uropathogens implicated in community-acquired UTI and their antimicrobial resistance pattern, when compared with UTI acquired at hospital level. This information is very important and reflects changes over the years, which implies a periodic monitorization in order to decrease the number of therapeutic failures [15,26,28].

The main objective of this study was to evaluate the prevalence and the antimicrobial resistance pattern of the main bacteria responsible for urinary tract infection in the community of Aveiro District (Portugal), throughout a ten year period, in order to establish an appropriate empirical therapy.

## Methods

### Samples

In this retrospective study, all urine samples from patients of the District of Aveiro, in ambulatory regime, collected at the Clinical Analysis Laboratory Avelab (Aveiro, Portugal) during the period 2000–2009 were analysed. Samples were collected from patients presenting clinical symptoms of urinary tract infection, from UTI post-treatment patients and pregnant women.

For each patient the collection date, age, sex, urine culture results, identification of the bacterial strain responsible for UTI and the correspondent Antimicrobial Susceptibility Test (AST) results were registered. The study was approved by the Ethical Committee of the Clinical Analysis Laboratory Avelab, specifically by Doctors Alberto Ferreira Neves, António Rodrigues and Maria Teresa Raposo.

### Sampling

Early urine sample was collected, using the Avelab Laboratory protocol, by midstream clean-catch technique after patient daily hygiene. The initial and the end portion of the micturition were discarded and the middle jact was collected directly into the sterile recipient. For children up to two years the urine sample was collected using a collection bag that was adhered to the skin surrounding the urethral area. The collection bag was controlled every fifteen minutes and after micturition the bag was removed, closed and stored at 4°C until processing. The urine samples were analyzed within one hour after collection. When this procedure was not possible the urine samples were stored at 4°C and processed until 24 h after collection.

### Microscopic examination

The samples of urine were homogenized and transferred to a conical tube of 10 mL. The urine was centrifuged at 2500 rpm for seven minutes and the supernatant was decanted. The pellet was homogenized and mounted on slides that were directly examinated or stained by the Gram technique.

### Urine culture

The urine samples were inoculated in different culture media. A calibrated loop of 1 μL was dipped in vertical position in the urine sample and the loop was used to inoculate the plates using the streak plate method. Gram negative bacilli, were detected using the Levine medium (Biokar Diagnostics, BK056HA). For Gram positive cocci, the urine samples were spread in Mannitol Salt Agar (Biokar Diagnostics, BK030HA) for the detection of *Staphylococcus spp*, in Bile Esculin Agar (BD BBL, 212205) for the detection of *E. faecalis* and in Blood Agar (Biomérieux, 43041) for the detection of *Streptococcus spp*. The Petri plates were incubated at 37°C during 24–72 hours, depending on the microorganism. The plates of Blood Agar were incubated in 5-10% CO_2_ atmosphere. After incubation, the urine cultures were classified as negative, positive and contaminated. The samples were classified as contaminated when polymorphic bacterial growth (growth of two or more bacterial species) was observed (exclusion criterium). The urine cultures were classified as negative when bacterial growth was lower than 10^3^ CFU/mL (exclusion criterium). When monomorphic bacterial growth was higher than 10^5^ CFU/mL the culture was classified as positive (inclusion criterium) and, for these cases, the AST was performed. The AST was also performed when the result of urine culture was between 10^4^ and 10^5^ CFU/mL.

### Identification of bacterial isolates

Additional biochemical tests were done when the urine culture was positive. These tests were performed based on the morphology of the isolated bacteria and on the results of the microscopic examination of the Gram-stained smear. The Enterobacteriaceae were differentiated using the the Kligler (BD BBL, 211317), Tryptone (BD BBL, 264410), Simmons Citrate (BD BBL, 211620) and Urea (Oxoid, CM0053) media. *Proteus mirabilis* was distinguished from the *Proteus vulgaris* by the indol test. The coagulase test (Biomérieux, Slidex Staph plus, 73115) was used to differentiate *Staphylococcus aureus* from the other *Staphylococcus*. *Staphylococcus epidermidis* (novobiocin-sensitive) was differentiated from *Staphylococcus saprophyticus* (novobiocin-resistant) using the novobiocin susceptibility test (BD BBL Sensi-Disc, 231314). The catalase test was used to distinguish *Staphylococcus spp* from *Enterococcus faecalis* and *Streptococcus spp*. The oxidase test (BD BBL, 231746) was used to identify Pseudomonaceae. The uropathogen *Pseudomonas aeruginosa* was identified by production of diffusible pigments on Mueller-Hinton Agar (Biokar Diagnostics, BK048HA) and for a grape-like odour released [29].

Reference strains *Escherichia coli* ATCC 25922, *Pseudomonas aeruginosa* ATCC 27853, *Staphylococcus aureus* ATCC 29123 and *Staphylococcus epidermidis* ATCC 12228 were used as positive control.

### Antimicrobial susceptibility test

The AST was performed using the modified Kirby-Bauer disk diffusion method. A bacterial suspension in physiological saline solution, with a turbidity of 0.5 on McFarland scale, was prepared by peaking up 1–2 colonies from pure cultures. The suspension was spread platted using a swab, on Mueller-Hinton Agar. Antimicrobial-impregnated disks were (BD BBL, Sensi-Disc) placed onto the cultures medium surface using an automated disk dispenser. For the Enterobacteriaceae, the antibiotics amoxicillin, cephradine, cefuroxime, amoxicillin-clavulanic acid, amikacin, gentamicin, ciprofloxacin, trimethoprim-sulfamethoxazole and nitrofurantoin were tested. For *Enterococcus spp* and *Streptococcus spp* penicillin, imipenem, amoxicillin-clavulanic acid, gentamicin, ciprofloxacin, nitrofurantoin and vancomycin were used. For *Staphylococcus spp*, penicillin, cephradine, amoxicillin-clavulanic acid, gentamicin, ciprofloxacin, nitrofurantoin and vancomycin. For *Pseudomonas spp* piperacillin, cefepime, aztreonam, imipenem, amikacin, gentamicin and ciprofloxacin were tested. The plates were incubated at 37°C for 18–24 hours. After incubation, the antimicrobials efficacy was determined by measuring the diameter of the zones of inhibition [30]. Bacterial strains were classified as susceptible (S), intermediate (I) or resistant (R) according the diameter of the inhibition zone [30].

### Statistical analysis

The data were treated using the Statistical Package for the Social Sciences (SPSS) 16.0 for Windows. The significant level established was 0.05. The normality of data, homogeneity and independence of variance were checked before analysis. As most of the variables failed these statistical method assumptions, the non-parametric Mann–Whitney and Kruskal-Wallis tests, were used. To simplify the statistical treatment it were selected the main bacteria responsible for UTI, namely *Escherichia coli, Staphylococcus aureus, Proteus mirabilis, Klebsiella spp, Enterococcus faecalis, Proteus vulgaris, Pseudomonas aeruginosa, Enterobacter spp, Staphylococcus epidermidis* and *Providencia spp*. All of these selected bacteria were responsible for at least 1% of the observed infections, which corresponded to 93.5% of all positives urine cultures. The bacteria responsible for less than 1% of UTI, account for 6.5% of observed infections.

To analyze the antibiotic resistance pattern of the main bacteria implicated in UTI, preliminary studies were performed to identify the most frequently used antimicrobials for each uropathogen, including in the study, the antimicrobials that were used in more than 85% of the cases. Uropathogens resistant to three or more antimicrobial classes were considered MDR [31].

## Results

From the 155597 samples analysed, 120691 (77.6%) were performed on female patients and 34898 (22.4%) on males, with an age range of 0 to 99 years. The middle-aged adults represented the age group that carry out more analysis, 54466 (35%), followed by young adults 51519 (33.1%), elderly patients 38455 (24.7%), children 7040 (4.5%) and adolescents 4117 (2.6%). From the 155597 urine samples, 18797 (12.1%) of the patients had urinary tract infection.

### Characterization of patients with bacterial UTI

The age of patients with bacterial UTI ranged from 3 to 99 years, with a mean age of 54 years. The average age of female patients with UTI was 51 years, lower than that of the males, 61 years. From the 18797 positive bacteriological tests, 4043 (21.5%) were from male patients and 14754 (78.5%) from female patients (Table 1). The elderly was the age group most affected by UTI with a frequency of 38.6% (27.3% corresponding to female and 11.3% to male patients) and the adolescents had the lowest frequency of UTI, 1.9% (1.6% for females and 0.3% for males). With the exception of the adolescents group, urinary tract infection increased with age (Table 1). A higher prevalence of infections in females for the all age groups was observed (Table 1).

**Table 1:** Incidence of the main ten bacteria implicated in urinary tract infection throughout the study period

	**Children**			**Adolescents**			**Young adults**			**Adults**			**Elderly**			**Isolates in the 10 years (%)**^**a**^	**Male (%)**^**a**^	**Female (%)**^**a**^	**MDR (%)**
	**0-12 years**			**13-18 years**			**19-34 years**			**35-64 years**			**> 65 years**						
**Bacteria**	**Total**^**a**^	**M**^**b**^	**F**^**b**^	**Total**^**a**^	**M**^**b**^	**F**^**b**^	**Total**^**a**^	**M**^**b**^	**F**^**b**^	**Total**^**a**^	**M**^**b**^	**F**^**b**^	**Total**^**a**^	**M**^**b**^	**F**^**b**^	**(N= 18797)**	**(N= 3641)**	**(N= 13939)**	
	**(N = 615)**	**(n =137)**	**(n=478)**	**(N= 340)**	**(n=46)**	**(n=294)**	**(N= 4113)**	**(n=322)**	**(n=3791)**	**(N= 5810)**	**(n=1240)**	**(n=4570)**	**(N= 6702)**	**(n=1896)**	**(n=4806)**				
*Enterobacter sp*	1.3	0.3	1.0	2.1	1.2	0.9^**c**^	2.0	0.3	1.7^**c**^	1.7	0.4	1.3	2.5^**d**^	1.3	1.1^**c**^	1.9	3.6^**c**^	1.7	60.8
*E. faecalis*	3.1	0.5	2.6	3.2	0.3	2.9	3.8	0.4	3.4	3.6	0.8	2.8	4.3	1.8	2.5^**c**^	3.6	5.2^**c**^	3.5	28.1
*E. coli*	71.1	15.8	55.3	65.9	5.9	60^**a**^	69.9	4.7	65.3^**c**^	71.4^**d**^	14.2	57.3^**c**^	66.2	14.9	51.4^**c**^	64.5	58.5	71.7^**c**^	17.0
*Klebsiella sp*	1.8	0.5	1.3	4.1	1.8	2.4^**a**^	3.1	0.4	2.7^**c**^	4.0	1.1	2.9^**c**^	6.3^**d**^	2.1	4.1^**c**^	4.3	6.3^**c**^	4.1	35.0
*P. mirabilis*	7.6^**d**^	2.8	4.9^**c**^	4.7	1.2	3.5	5.2	0.7	4.6^**c**^	4.7	1.0	3.7	4.8	1.6	3.3	4.7	5.8^**c**^	4.8	50.3
*P. vulgaris*	2.4	0.8	1.6	3.2	0.6	2.6	2.5	0.2	2.3	2.6	0.8	1.8^**c**^	3.3	0.9	2.3	2.7	3.5^**c**^	2.7	95.4
*Providencia sp*	2.0	0.3	1.6	2.1	0.3	1.8	1.8	0.1	1.6	1.7	0.3	1.4	1.9	0.6	1.3	1.7	1.8	1.8	95.3
*P. aeruginosa*	1.5	0.2	1.3	1.5	0.3	1.2	1.3	0.3	0.9^**c**^	1.8	0.9	0.9^**c**^	4.2^**d**^	2.2	2.0^**c**^	2.4	5.9^**c**^	1.7	24.7
*S. aureus*	5.5	0.8	4.7	11.5^**d**^	1.8	9.7	8.2	0.6	7.6	6.5	1.4	5.1	5.0	2.2	2.8^**c**^	6.0	7.2^**c**^	6.2	18.8
*S. epidermidis*	3.7^**d**^	0.3	3.4	1.8	0.3	1.5	2.1	0.1	2.0	2.0	0.5	1.5	1.6	0.7	0.9^**c**^	1.8	2.1	1.9	22.9
**Total of UTI (%)**	3.5	0.8	2.7	1.9	0.3	1.6	23.1	1.9	21.2	32.8	7.2	25.6	38.6	11.3	27.3		21.5	78.5	

The incidence of UTI by sex, age group and multidrug resistance percentage.

*N* total number of bacteria for each age group; n: total number of bacteria for each sex; ^a^Percentage determined in relation to N; ^b^Percentage determined in relation to n; ^**c**^statistically significant differences of frequency between sex; ^**d**^statistically significant differences between age groups; *M* male, *F* female, *MDR* multidrug resistant.

### Bacteria implied in UTI

The ten bacteria more implicated in the UTI (each of them implicated at least in 1% of the UTI) in the period of ten years, represented annually more than 90% of the bacterial isolates. In 2001, the main bacteria reached the higher percentage, representing 98.6% of the total and in 2005 the lowest percentage, representing 91.6% of the isolates.

The predominant agents of UTI were successively *Escherichia coli* (64.5%), *Staphylococcus aureus* (6.0%), *Proteus mirabilis* (4.7%), *Klebsiella spp* (4.3%), *Enterococcus faecalis* (3.6%), *Proteus vulgaris* (2.7%), *Pseudomonas aeruginosa* (2.4%), *Enterobacter spp* (1.9%), *Staphylococcus epidermidis* (1.8%) and *Providencia spp* (1.7%) (Table 1).

**Figure 1:**
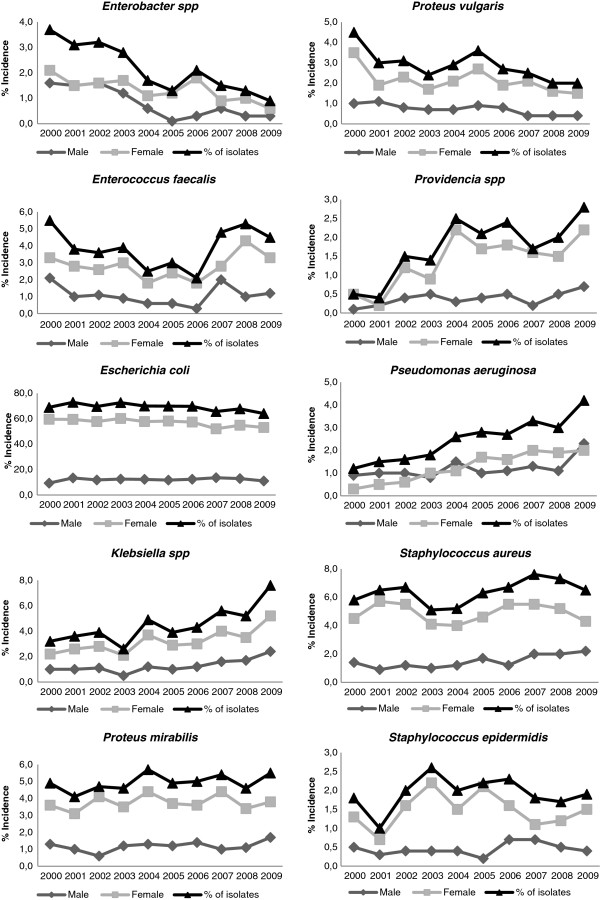
Incidence of the main bacteria implicated in UTI by sex during the study period.

Significant (Kruskal-Wallis test, ρ < 0.05) changes in the main bacteria responsible for UTI were observed during the study period. In general, the incidence of *P. aeruginosa*, *Providencia spp* and *Klebsiella spp* increased and the incidence of *Enterobacter spp* decreased (Figure 1). Despite the differences in bacterial etiology along the year period, *E. coli* was the pathogen most implicated in UTI throughout all the study period, followed by *S. aureus*.

The incidence of the bacteria implicated in UTI in women was statistically different (Mann–Whitney U test, p < 0.05) from that causing UTI in men. Although the bacterium *E. coli* was the most implicated in UTI in both sexes, its incidence was higher in female patients (average 71.7%) than in males (average 58.5%) (Table 1). The incidence of *Pseudomonas aeruginosa* was also different for male and female patients, corresponding to the fourth cause of UTI in males and the ninth cause in females, causing 5.9% and 1.7% of the UTI, respectively (Table 1).

The incidence of the ten main bacterial uropathogens throughout the study period was, more stable for male than for female patients and was, in general, higher in the female patients (Figure 1). However, the incidence of *P. aeruginosa* in males was frequently higher than in female patients. The incidence of *Providencia spp* and of *Enterobacter spp* in the period 2001–2002 was similar in both sexes.

The most implicated bacteria in UTI in the different age groups were not statistically different (Kruskal-Wallis test, p> 0.05) when all the samples were considered (female and males samples). However, significant differences (Mann–Whitney U test, p < 0.05) of bacterial aetiology for all age groups were observed when females and males were analyzed separately, increasing the significance of the difference with the patient age (Table 1).

### Antimicrobial resistance pattern of the main bacteria implicated in UTI

The Gram negative bacteria showed higher resistance to penicillins, quinolones and first-generation cephalosporins than to the other tested antimicrobials (Table 2). *E. coli* was the uropathogen that showed the lowest resistance to antimicrobials (Table 2). The isolates of *Providencia spp*, *Proteus vulgaris* and *Enterobacter spp* showed high resistance to several of the tested antimicrobials (Table 2). The Gram positive bacteria showed also higher resistance to penicillins and quinolones than to the other tested antibiotics. For the other antimicrobial groups resistance was less than 14%. For glycopeptides, carbapenems and nitrofurans the resistance was lower than 1.5% (Table 3). The isolates of *S. aureus* showed high resistance to penicillin (average of 55.1%) and to the quinolone lomefloxacin (average of 21.1%) (Table 3). The isolates of *E. faecalis* were the Gram positive bacteria with the highest resistance to the tested antimicrobials (Table 3).

**Table 2:** Averaged antimicrobial resistance of the main Gram negative uropathogens for female and male patients

**Antimicrobial group**	**Antimicrobials**	***E. coli***				***P. mirabilis***				***Klebsiella spp***				***P. vulgaris***				***P. aeruginosa***				***Enterobacter spp***				***Providencia spp***			
		**N**	**%**	**M**	**F**	**N**	**%**	**M**	**F**	**N**	**%**	**M**	**F**	**N**	**%**	**M**	**F**	**N**	**%**	**M**	**F**	**N**	**%**	**M**	**F**	**N**	**%**	**M**	**F**
Aminoglycosides	Amikacin	12048	1.5	1.9	1.4	865	4.3	5.7	3.8	800	2.6	2.6	2.6	492	7.3	6.4	7.6	443	7.9	7.1	8.7	360	7.8	9.1	7.0	307	92.8	89.2	93.8
	Gentamicin	12034	3.6	6.3*	3.1	868	5.4	7.1*	4.9	798	7.3	8.4	6.8	487	12.9	12.2	13.2	441	15.2	15.7	14.7	361	10.8	13	9.6	307	93.8	90.8	94.6
	Isepamicin	10995	1.3	1.8	1.3	787	3.4	4.6	3.1	707	2.0	2.4	1.8	444	6.3	5.4	6.6	387	8.3	8.2	8.3	348	3.2	4.1	2.7	282	92.9	91.7	93.2
	Netilmicin	11976	1.3	1.7	1.2	854	0.9	2.9*	0.3	792	2.5	2.7	2.5	484	5.8	7.3	5.3	443	14.4	15.6	13.4	358	8.7	14.5*	5.3	307	94.5	93.8	94.6
	Tobramycin	11999	3.0	4.9*	2.6	860	4.1	7.3*	3.1	793	5.8	8.0*	4.9	494	12.6	14.4	11.9	443	16.5	17.0	16.0	358	14.5	23.1*	9.6	306	94.4	93.8	94.6
Carbapenems	Imipenem	11846	0.2	0.4	0.1	846	1.3	2.9*	0.8	785	0.4	0.0	0.5	482	2.9	2.4	3.1	425	2.4	2.0	2.7	337	2.1	4.3*	0.9	308	1.3	3.2	0.8
Cephalosporins 1^st^ G	Cefazolin	11919	10.5	15.8*	9.4	862	22.2	31.1*	19.3	766	29.9	37.3*	26.9	484	94.8	97.6	93.8	152	98.0	97.3	98.7	346	96.0	97.6	95.0	311	95.5	92.4	96.3
	Cephradine	11994	14.1	19.2*	13.0	862	22.3	30.8*	19.5	788	32.0	38.1	29.6	486	95.9	98.4	95.0	155	98.7	98.7	98.8	360	96.4	97.7	95.6	310	96.8	95.3	97.2
Cephalosporins 2^nd^ G	Cefoxitin	11954	5.1	7.2*	4.7	860	15.8	20.2*	14.4	781	19.0	23.1	17.3	489	26.8	33.9	24.4	170	97.6	98.8	96.6	353	64.0	74.6*	58.1	308	8.4	19.0*	5.7
	Cefuroxime	11933	4.3	6.7*	3.8	863	9.6	12.9*	8.6	781	16.1	22.0*	13.8	481	25.4	32.5*	22.9	165	98.8	98.8	98.8	356	62.4	75*	55.3	317	10.1	24.2*	6.4
Cephalosporins 3^rd^ G	Cefodizime	8959	0.4	0.8	0.3	609	1.3	1.3*	1.3	595	2.4	4.2	1.6	320	4.1	3.8	4.1	311	7.7	6.4	8.8	253	8.7	13.5	6.1	269	0.0	0.0	0.0
	Ceftazidime	11743	1.2	1.6	1.1	863	5.9	4.8	6.3	776	7.9	12.6*	6.0	487	10.3	12.1	9.6	436	4.1	1.9	6.1	347	13.8	17.6	11.7	310	1.0	1.6	0.8
	Ceftibuten	12022	0.9	1.3	0.8	864	3.8	3.9	3.8	791	2.4	1.8	2.6	485	5.8	6.7	5.5	425	26.8	28.2	25.6	355	11.5	15.1*	9.6	313	0.0	0.0	0.0
Cephalosporins 4^th^	Cefepime	11921	0.3	0.3	0.3	859	3.7	2.4	4.1	763	1.6	2.8	1.1	488	4.1	4.8	3.8	420	4.8	5.0	4.5	349	5.7	8.7*	4.0	314	0.0	0.0	0.0
Monobactams	Aztreonam	11922	2.9	3.5	2.8	861	15.4	14.6	15.7	775	6.2	6.8	5.9	470	21.5	24.2	20.6	423	5.4	5.5	5.4	329	19.8	19.3	20.0	310	5.2	6.3	4.9
Nitrofurans	Nitrofurantoin	11768	6.0	9.0*	5.4	863	95.1	95.7	95.0	768	32.8	39.5*	30.2	483	97.1	100.0*	96.1	—	—	—	—	347	39.5	51.6*	32.6	307	87.3	89.2	86.8
Penicillins	Amoxicillin	11943	42.4	50.3*	40.8	863	47.7	55.3*	45.3	372	96.8	99.0	95.9	487	97.9	99.2	97.5	—	—	—	—	359	98.6	99.2	98.3	314	99.0	100.0	98.8
	AMX-CLA	11879	7.5	10.3*	6.9	863	11.8	20.1*	9.2	316	81.6	85.1	80.3	486	32.9	41.9*	29.8	—	—	—	—	345	83.2	87.1	81.0	309	18.4	27.7*	16.0
	Piperacillin	11749	19.5	24.0*	18.5	832	20.0	24.7	18.5	—	—	—	—	457	37.4	49.6*	33.4	418	24.2	24.1	24.2	335	34.9	45.8*	29.0	300	26.7	33.8	24.7
	Pivmecillinam	11546	15.8	20.9*	14.7	812	37.7	44.7*	35.4	707	24.8	32.4*	21.5	450	59.1	71.9*	54.8	—	—	—	—	323	46.7	57.1*	41.2	285	24.2	32.8*	22.0
Quinolones	Ciprofloxacin	11952	13.9	22.2*	12.2	865	21.3	35.1*	16.9	784	22.3	32.4*	18.3	484	25.0	36.4*	21.2	440	43.4	45.7	41.4	357	23.8	36.9*	16.3	312	18.3	27.7*	15.8
	Lomefloxacin	10634	13.7	22.6*	11.9	765	21.8	33.0*	18.5	641	22.9	34.1*	18.8	420	25.2	38.8*	20.8	349	43.8	46.9	41.2	302	25.2	44.7*	15.1	272	18.8	28.3*	16.4
	Norfloxacin	11893	14.2	22.8*	12.3	867	22.8	36.2*	18.6	779	21.7	31.3*	18.0	470	24.9	36.2*	21.2	437	44.9	48.1	42.0	355	26.5	44.2*	16.4	307	19.2	29.7*	16.5
	Ofloxacin	11937	14.4	23.0*	12.6	860	22.9	35.9*	18.7	783	22.2	32.3*	18.2	483	26.1	38.2*	21.9	438	45.0	48.3	42.1	356	27.2	44.6*	17.3	310	19.4	29.7*	16.7
Sulfonamides	SxT	11907	25.4	33.0*	23.8	831	41.5	55.0*	37.2	776	36.0	41.4	33.8	482	57.3	64.5	54.7	—	—	—	—	356	39.3	56.6*	29.5	303	23.1	30.3	21.1

*AMX - CLA* Amoxicillin-clavulanic acid, *SxT* Trimethoprim-Sulfamethoxazole, *n* total number of bacteria resistant to each antimicrobial, *N*: total number of bacteria tested against each antimicrobial; — Antimicrobial not tested, *M* male patient, *F* female patient, *statistically significant differences (p-value < 0.05) of antimicrobial resistance between female and male patients.

**Table 3:** Averaged antimicrobial resistance of the main Gram positive uropathogens for female and male patients

**Antimicrobials group**	**Antimicrobials**	***S. aureus***				***E. faecalis***				***S. epidermidis***			
		**N**	**%**	**M**	**F**	**N**	**%**	**M**	**F**	**N**	**%**	**M**	**F**
Aminoglycosides	Amikacin	1104	5.5	5.8	5.4	—	—	—	—	336	5.1	1.3	6.2
	Gentamicin	1116	6.2	7.3	5.9	652	31.7	35.6	30.3	337	5.6	7.7	5.0
	Isepamicin	1023	5.4	5.5	5.3	447	62.9	67.2	61.2	314	1.3	0.0	1.6
	Netilmicin	1094	3.3	4.3	3.0	—	—	—	—	336	1.5	0.0	1.9
	Tobramycin	1107	6.4	8.1	5.9	—	—	—	—	335	6.6	9.0	5.8
Carbapenems	Imipenem	1106	2.1	3.9	1.5	672	6.3	5.2	6.7	336	0.0	0.0	0.0
Cephalosporins 1^st^ G	Cefazolin	1097	15.6	22.0	13.6	—	—	—	—	324	12.0	10.5	12.5
	Cephradine	1083	16.5	24.1*	14.2	—	—		—	334	12.3	11.7	12.5
Cephalosporins 2^nd^ G	Cefoxitin	1097	16.6	20.2	15.5	—	—	—	—	337	13.1	9.1	14.2
	Cefuroxime	1091	15.1	20.7	13.4	—	—	—	—	340	13.5	7.7	15.3
Cephalosporins 3^rd^ G	Cefodizime	841	10.8	12.8	10.2	—	—	—	—	245	8.2	6.8	8.6
	Ceftazidime	1084	17.1	19.0	16.5	—	—	—	—	336	18.8	11.7	20.8
	Ceftibuten	1088	11.1	14.9	10.0	—	—	—	—	339	13.6	7.8	15.3
Cephalosporins 4^th^ G	Cefepime	1086	10.7	14.2	9.6	—	—	—	—	338	11.8	9.1	12.6
Glycopeptides	Teicoplanin	827	3.1	4.4	2.7	621	4.0	5.7	3.4	233	0.9	0.0	1.1
	Vancomycin	777	2.6	3.6	2.2	472	1.1	0.0	1.4	218	0.0	0.0	0.0
Nitrofurans	Nitrofurantoin	—	—	—	—	651	8.0	7.9	8.0	—	—	—	—
Penicillins	AMX-CLA	1107	8.3	15.1*	6.3	665	9.5	9.6	9.4	338	3.0	1.3	3.5
	Flucloxacillin	992	16.8	20.3	15.8	—	—	—	—	304	15.8	12.3	16.9
	Penicillin	1036	55.1	64.5	52.2	646	59.8	61.6	59.1	315	58.1	45.9	61.8
Quinolones	Ciprofloxacin	1103	18.0	33.3*	13.4	260	38.1	53.9*	31.5	331	26.6	32.9	24.8
	Lomefloxacin	845	21.1	36.4*	15.8	206	45.1	58.9*	40.0	254	26.4	32.8	24.4
	Norfloxacin	1073	18.3	32.9*	13.8	262	40.8	50.0	37.4	326	25.8	30.3	24.4
	Ofloxacin	1091	17.8	31.9*	13.5	265	40.4	48.6	37.2	333	25.5	30.3	24.1
Sulfonamides	SxT	1075	15.6	22.4*	13.5	609	35.5	45.3*	31.6	328	25.9	29.7	24.8

*AMX - CLA* Amoxicillin-clavulanic acid, *SxT* Trimethoprim-Sulfamethoxazole, *n* total number of bacteria resistant to each antimicrobial, *N* total number of bacteria tested against each antimicrobial; — Antimicrobial not tested, *M* male patient, *F* female patient, * statistically significant differences (p-value < 0.05) of antimicrobial resistance between female and male patients.

**Figure 2:**
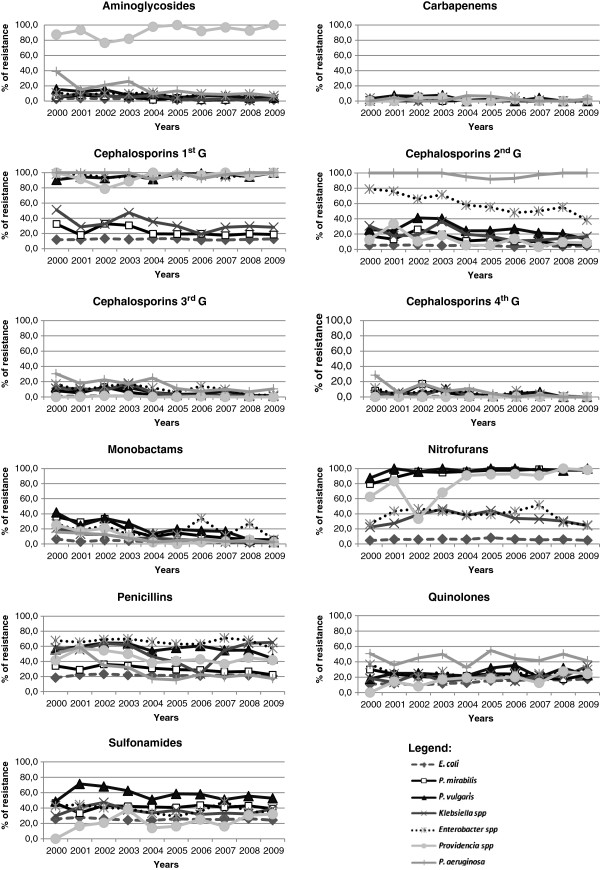
Variation of antimicrobial resistance pattern of Gram negative bacteria during the study period.

**Figure 3:**
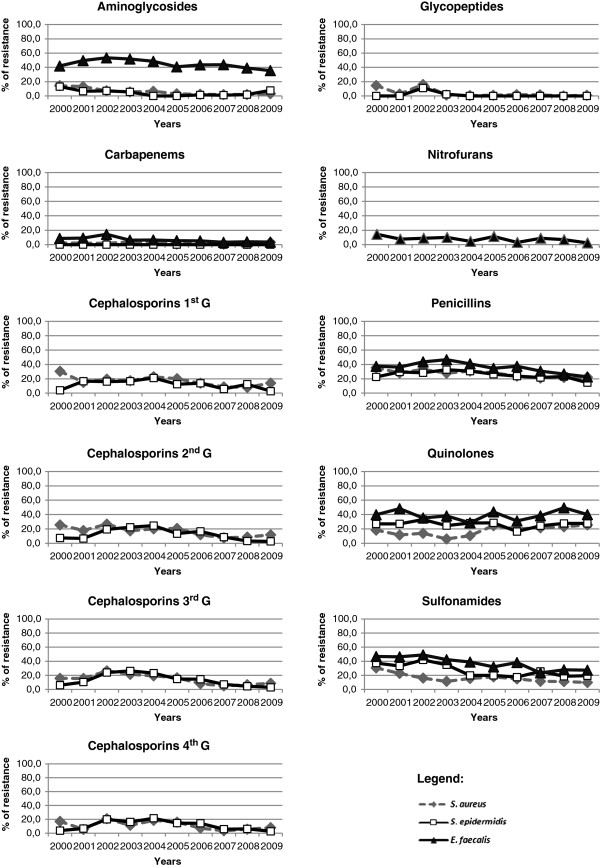
Variation of antimicrobial resistance pattern the Gram-positive bacteria during the study period.

The bacterial resistance, in general, changed significantly over the study period (Kruskal-Wallis test, p < 0.05) but *E. coli* did not show significant changes in the resistance to the tested antimicrobials (Figure 2). The pattern of resistance of the Gram negative Enterobacteriaceae along the study period was different from that observed for *P. aeruginosa* (Figure 2). The three Gram positive bacteria showed a decrease in the resistance to the antibiotic penicillin and to sulfonamide SXT (Figure 3). For the two *Staphylococcus* it was observed also a decrease in the resistance to the four generations of cephalosporins (Figure 3).

The resistance of the isolates most implicated in UTI in male patients was statistically different (Mann–Whitney U test, p< 0.05) from that of female patients. Bacteria isolated from female patients were, on average, resistant to less than 3 antimicrobials, while the bacteria isolated from male patients were, on average, resistant to 4 antimicrobials. However, for *S. epidermidis* and *P. aeruginosa*, no significant differences between sexes were observed (Tables 2 and 3).

In general, an increase of the bacterial resistance was observed with the age (Kruskal-Wallis test, p < 0.05). For *E. coli*, the resistance to the two antibiotics of first line choice for uncomplicated UTI, tested in this study was low, 6% for nitrofurantoin and 15.8% for pivmecillinam. For the other bacteria implicated in UTI, the resistance to these two first line antibiotics was higher than that observed for E. coli with an average of 60% for nitrofurantoin and 39% for pivmecillinam. Having into account the incidence and the values of drug resistance of each bacterium, it was calculated the pondered resistance patterns according to the uropathogens incidence (multiplying the bacterium averaged resistance by its incidence) for the two first line antibiotics indicated to treat UTI (Table 4). The obtained values were 16% and 15%, respectively, for pivmecillinam and nitrofurantoin (Table 4).

**Table 4:** Pondered bacterial resistance to the antimicrobials recommended for empirical treatment of urinary tract infection

		**Resistance to first-line therapy**					**Resistance to second-line therapy**				
**Bacteria**	**Incidence (%)**	**PIV (%)**	**PIV (%)**^**1**^	**NIT (%)**	**NIT (%)**^**1**^	**AMX-CLA (%)**	**AMX-CLA (%)**^**1**^	**SXT (%)**	**SXT (%)**^**1**^	**FLU (%)**	**FLU (%)**^**1**^
*E. coli*	64.5	15.8	10.2	6.0	3.9	7.5	4.8	25.4	16.4	14.1	9.1
*S. aureus*	6.0	—	—	—	—	8.3	0.5	15.6	0.9	18.8	1.2
*P. mirabilis*	4.7	37.7	1.8	95.1	4.5	11.8	0.6	41.5	2.0	22.2	1.1
*Klebsiella spp*	4.3	24.8	1.1	32.8	1.4	81.6	3.5	36.0	1.5	22.3	1.0
*E. faecalis*	3.6	—	—	8.0	0.3	9.5	0.3	35.5	1.3	41.1	1.5
*P. vulgaris*	2.7	59.1	1.6	97.1	2.6	32.9	0.9	57.3	1.5	25.3	0.7
*P. aeruginosa*	2.4	—	—	—	—	—	—	—	—	44.3	1.1
*Enterobacter spp*	1.9	46.7	0.9	39.5	0.8	83.2	1.6	39.3	0.7	25.7	0.5
*Snn epidermidis*	1.8	—	—	—	—	3.0	0.1	25.9	0.5	26.1	0.5
*Providencia*	1.7	24.2	0.4	87.3	1.5	18.4	0.3	23.1	0.4	18.9	0.3
Pondered Resistance (%)			15.9		14.9		12.6		25.2		16.8

The pondered bacterial resistance was determined having into account the incidence and resistance of each bacterium. For the fluoroquinolones the pondered bacterial resistance by sex was also determined (25.6% for males and 14.3% for females).

Relatively to the alternative drugs, the resistance of *E. coli* was also low, 14% and 8%, respectively, for the fluoroquinolones and AMX-CLA, but for the SXT the resistance was higher 25%. The other bacteria presented high values of resistance for these alternative antibiotics, average of 27%, 31% and 34%, respectively, for fluoroquinolones, AMX-CLA and SXT. Considering the incidence of each bacterium and the values of its resistance to antibiotics, the pondered resistance was 17%, 13% and 25%, respectively, for fluoroquinolones, AMX-CLA and SXT (Table 4). For the fluoroquinolones the pondered resistance was significantly higher (Mann–Whitney U test, p < 0.05) for male than for female (pondered resistance 26% and 14% for male and female, respectively).

### Multidrug resistant bacteria implicated in UTI

The percentage of MDR isolates varied between 17 and 95.4%. *P. vulgaris* and *Providencia spp* were the most common MDR uropathogens (Table 1). The bacteria more implicated in UTI, *E. coli* and *S. aureus*, showed the lowest percentage of MDR isolates (17.0 and 18.8%, respectively) (Table 1). Significant differences (Kruskal-Wallis test, p < 0.05) in bacterial multidrug resistance were observed throughout the study period. The MDR bacteria isolates from female patients were statistically different (Mann–Whitney U test, p < 0.05) from that observed in bacteria isolated from male patients. The most implicated bacteria in UTI were, on average, resistant to a higher number of antimicrobials classes when isolated from male patients than from female ones. The incidence of MDR bacteria were statistically different (Kruskal-Wallis test, p < 0.05) among the different age groups, in general, an increase in resistance and in MDR with the patient age was observed.

## Discussion

Although *E. coli* was the most frequent uropathogen implicated in community-acquired UTI (being implicated in more than an half of all the UTI), as frequently detected in other studies [10,13,16,17], significant differences in relation to the other bacteria involved in the community-acquired UTI were observed relatively to other studies performed at the community level. Contrary to other studies, *S. aureus* was the second most frequent uropathogen involved in the UTI. Even though *S. aureus* has been associated to hospitalized patients that have undergone catheterization and may be associated to urinary tract infections [32,33], this bacterium has appeared with high frequency in the community in individuals who were not hospitalized or underwent medical procedures such as dialysis, surgery or catheters. An example is the patients with atopic dermatitis who are usually colonized by *S. aureus*, due to changes in the first line of defence. These individuals serve as major vectors for its transmission [34,35]. In 1997 it was reported that 29% of healthy adults outside the hospital environment are colonized by MRSA. Today, it is known that this value increased to 74% [36]. This type of colonization is caused by strains of *S. aureus* different from those found in the hospital environment and are often referred as community-associated MRSA (CA-MRSA). Some studies have shown that CA-MRSA has high potential to become endemic in the community and that this will have a significant impact on the control of MRSA in hospitals [37-39]. Portugal is one of the European countries with MRSA rates higher than 50% [36] which may explain the higher frequency of this bacterium in UTI at the community level relatively to other countries. *Staphylococcus saprophyticus*, according to the literature has been the second most common cause of uncomplicated UTI, causing 5-10% of the UTI, [40,41] but in this study *S. saprophyticus* was not among the most implicated bacteria in UTI.

Although *E. coli* was the most common uropathogen in both sexes, its incidence was significantly higher in women (71.7%) than in men (58.5%). However, *P. aeruginosa* was the uropathogen more responsible for the differences between female and male patients, emerging as the fourth and ninth causes of infection, respectively. According to the literature, *P. aeruginosa* is more frequent in males due to particular characteristics inherent to the patient, including sex, previous use of antimicrobials, previous interventions in the urinary tract and patients with neurogenic bladder [42].

Even though it has been stated that factors such as age might influence the aetiology of urinary tract infection, [14,43,44] in this study it were not observed significant differences among the bacteria responsible for these infections in the different age groups when all samples were considered. However, significant differences were observed for all age groups when female and male were analysed separately and, the differences increasing with the patient age.

The incidence of the two bacteria more implicated in UTI was stable during the study period, but the incidence of some bacteria less implicated in UTI changed significantly, *P. aeruginosa*, *Providencia spp* and *Klebsiella spp* increased and *Enterobacter spp* decreased. The increase in the incidence of *P. aeruginosa* (four times during the study period) seems to be related with the increase of the patient average life expectancy, which potentially increases the hospitalization of elderly patients, increasing the transmission of bacterial strains between hospital and community, and not to the increase of bacterial resistance to the antimicrobials. In fact, the increase in *P. aeruginosa* was significantly higher for the elderly group during the study period. Despite community-acquired urinary infections caused by *P. aeruginosa* are still uncommon, this bacterium should not be ignored, since its incidence is increasing and this pattern of variation has been also observed in other community-acquired UTI studies [11,45]. The increase in the incidence of *Providencia spp* (five times during the study period) contrarily to *P. aeruginosa*, seems to be related with its antibiotic resistance increase. Although infections caused by this uropathogen are less frequent, in recent years they have increased essentially due to the production of extended spectrum beta lactamases (ESBL) [46]. In fact, in this study it was observed an increase over the ten years in the resistance of *Providencia spp* to nitrofurantoin, fluoroquinolones and SXT, all antibiotics used to treat UTI. The increase in the incidence of this bacterium does not seem to be related to the increase of elderly patient hospitalization as its incidence was similar for all the age groups during the study period. The incidence of *Klebsiella spp* doubled during the study period, being this increase more accentuated in the last three years when its resistance to AMX-CLA and quinolones also increased. The enhancement can be also related with the average life expectancy increase as *Klebsiella spp* was more frequently isolated in elderly UTI patients as in other community UTI studies [47,48]. The decrease of *Enterobacter spp* incidence (four times) can be related with its antimicrobial resistance decrease. Another study performed in the community during a period of seven years showed, however, that antimicrobial resistance of *Enterobacter spp* was stable over the years [49].

Although *E. coli* was responsible for more than half of the UTI, its antimicrobial resistance was significantly lower than that presented by the other bacteria less implicated in UTI. Having into account the incidence of each bacterium (65% for *E. coli* and 29.1% for the other bacteria) both first line antibiotics choice for uncomplicated UTI tested in this study (nitrofurantoin and pivmecillinam) drugs are suitable for UTI treatment in the community. Relatively to the alternative therapy drugs, considering the incidence of each uropathogen, according to the EAU, the AMX-CLA is appropriate to treat UTI in the community but fluoroquinolones should not be suitable to treat male infections and the SXT should be unsuitable to treat even female infections as the resistance was higher than the recommended value (<20%) indicated.

The results of antimicrobial resistance indicate that the choice of empirical antimicrobial therapy should have into account the sex of the patient. The uropathogens isolated from male patients presented, on average, resistance to a higher number of antibiotics (on average 4) than those isolated from female patients (on average less than 3). This difference was clearly evident for *E. coli, P. mirabilis* and *Enterobacter spp* which presented significant differences for at least 15 of the 25 antimicrobials tested (corresponding to 60-70% of the antibiotics). For the other three Enterobacteriaceae and for *E. faecalis*, the difference was also evident (at least for 39% of the tested antibiotics). The difference between male and female was clearly evident (with the exception of *P. aeruginosa* and *S. epidermidis*) for the fluoroquinolones indicated by the EAU as alternative therapy drug. Consequently this antibiotic should be suitable to treat female infections (resistance <20% for 70% of the bacteria more implicated in UTI) but should be not appropriate to treat male infection (resistance >20% for all of the ten bacteria more implicated in UTI).

For *P. aeruginosa* and *S. epidermidis*, however, it was not observed significant differences of bacterial resistance between sexes for the antibiotics tested, probably due to the frequent association of these two bacteria with hospital-acquired infections. Although the pattern of resistance of *P. aeruginosa* was not different for both sexes, as this bacterium is a more important cause of community-acquired UTI in men than in women, the empiric therapy must consider the clinical history for male patients, such as recent hospitalizations, in order to evaluate if UTI can be due *P. aeruginosa*, and, in these cases, the fluoroquinolones must be avoided. In a similar study done in the community, the bacterial resistance of *E. coli*, *Klebsiella spp*, *Proteus spp*, *Citrobacter spp* and *Enterobacter spp* were, in general, higher when the uropathogens were isolated from male patients [50]. However, Koeijers et al. [42] showed that the uropathogens responsible for community-acquired urinary tract infections, namely caused by *E. coli*, *P. mirabilis* and *K. pneumoniae* had similar susceptibility patterns in females and males.

In general, it was observed an increase of the bacterial resistance with the age of the patients. The uropathogens isolated from the elderly group were more resistant than the isolates from other age groups, which may be explained by the increase of the number and the duration of hospital admissions with the increasing age of the patients.

Whereas, in general, the first line drugs and at least one of the alternative drug used to treat non-complicated infections, are appropriate for treat community-acquire UTI which are usually treated at home, to treat more complicated infections caused, for instance by MDR strains (frequent in this study), a tailored therapy will be necessary. For these cases, the carbapenem imipenem, the third and fourth generation chephalosporins, aminoglycosides and glycopeptides (for Gram negative bacteria) should be suitable as the bacteria more implicated in community-acquired UTI do not present high resistance to these drugs. However, most of these drugs do not exist in the oral form, involving the inconvenience of parenteral administration, and are also more expensive than those indicated for the UTI treatment.

## Conclusions

As with the age increase, the differences in the uropathogen incidence between male and female patients increase and the bacteria isolated from male were, in general, more resistant to antibiotics when compared with bacteria isolates from female patients, the choice of empirical antimicrobial therapy should have into account the sex of the patients.

The results obtained in this study suggest the first line drugs indicated to treat non-complicated UTI (pivmecillinam and nitrofurantoin) as well as the alternative antibiotic AMX-CLA are appropriate drugs but the fluoroquinolones should not be suitable to treat male infections and the SXT antibiotic should not be even be considered to treat female UTI.

Considering that in this study (1) *S. aureus* was more frequent than in other international studies, (2) the bacterium *S. taphylococcus saprophyticus* was less frequent, (3) the resistance of the main bacterium implicated in UTI, *E. coli*, to the fluoroquinolone ciprofloxacin and to nitrofurantoin was higher than that observed in other European countries and (4) the bacteria isolated from male patients were more resistant to most of the antibiotics indicated for UTI treatment than those isolated from females (pattern of variation similar to that observed in some countries but different from that found in others), the results of this study cannot be translated to the international level, confirming the statement of the World Health Organization that local data on antimicrobial incidence and resistance are essential to define the best treatment for individual patients, to formulate recommendations for rational antibiotic use and standard treatment guidelines, ensuring that health-care providers comply with recommendations.

## Abbreviations

UTI: Urinary tract infection; CFU: Colony forming units; rpm: Rotations per minute; SXT: Trimethoprim-Sulfamethoxazole; AMX-CLA: Amoxicillin-Clavulanic Acid; EAU: European association of urology; MDR: Multidrug resistance; AST: Antimicrobial susceptibility test; SPSS: Statistical package for the social sciences; ESBL: Extended spectrum beta lactamases; ARESC: Antimicrobial resistance epidemiological survey on cystitis.

## Competing interests

The authors declare that they have no competing interests.

## Authors’ contributions

IL did the statistical analysis of data and drafted the manuscript. AA has been involved in the coordination, conception, design of the study and helped to draft the manuscript. TR and AR participated in the design of the study, acquisition and interpretation of data, and also helped to draft the manuscript. All authors have read and approved the final manuscript.

## Pre-publication history

The pre-publication history for this paper can be accessed here:

http://www.biomedcentral.com/1471-2334/13/19/prepub
